# Triglyceride-glucose index as a predictor of obstructive sleep apnoea severity in the absence of traditional risk factors

**DOI:** 10.1055/s-0043-1776411

**Published:** 2023-11-08

**Authors:** Sinem Nedime Sökücü, Şenay Aydın, Celal Satıcı, Seda Tural Önür, Cengiz Özdemir

**Affiliations:** 1 University of Health Sciences, Yedikule Chest Disease and Thoracic Surgery Training and Research Hospital, Sleep Laboratory, Istanbul, Turkey.; 2 Liv Hospital, Vadi Istanbul, Istanbul, Turkey.

**Keywords:** Sleep Apnea, Obstructive, Prognosis, Triglyceride Glucose Index, Apneia Obstrutiva do Sono, Prognóstico, Índice Triglicerídeos e Glicose

## Abstract

**Objective** We evaluated the association between the triglyceride–glucose (TG) index, a marker of insulin resistance, and obstructive sleep apnoea (OSA) severity in patients without diabetes mellitus, obesity, and metabolic syndrome.

**Methods** This retrospective cohort study included 1,527 patients. We used univariate and multivariate analyses to identify the independent predictors associated with OSA.

**Results** Most patients were males (81.5%) with a mean age of 43.9 ± 11.1 (15–90) years. Based on the apnoea–hypopnea index (AHI), 353 (23.1%) patients were included in the control group, whereas 32.4%, 23.5%, and 21% had mild, moderate, and severe OSA, respectively. The TG index values demonstrated significant associations with OSA patients compared with the control group ( *p* = 0.001). In addition, the mean values of the oxygen desaturation index (ODI), AHI, minimum oxygen saturation, and total sleep time percentage with saturation below 90% demonstrated statistically significant differences among the TG index groups (p: 0.001; p:0.001; p:0.001; p:0.003). The optimal TG index cutoff value to predict OSA was 8.615 (AUC = 0.638, 95% CI = 0.606–0.671, *p* = 0.001). In multivariate logistic regression analysis, after adjusting for age, sex, and body mass index, the TG index was independently associated with OSA patients.

**Conclusion** The TG index is independently associated with increased risk for OSA. This indicates that this index, a marker for disease severity, can be used to identify severe OSA patients on waiting lists for PSG.

## Resumo

**Objetivo** Avaliamos a associação entre o índice triglicerídeos-glicose (TG), um marcador de resistência à insulina, e a gravidade da apneia obstrutiva do sono (AOS) em pacientes sem diabetes mellitus, obesidade e síndrome metabólica.

**Métodos** Este estudo de coorte retrospectivo incluiu 1.527 pacientes. Utilizamos análises univariadas e multivariadas para identificar os preditores independentes associados à AOS.

**Resultados** A maioria dos pacientes era do sexo masculino (81,5%) com idade média de 43,9 ± 11,1 anos (15-90). Com base no índice apneia-hipopneia (IAH), 353 (23,1%) pacientes foram incluídos no grupo de controle, enquanto 32,4%, 23,5% e 21% tinham AOS leve, moderada e grave, respectivamente. Os valores do índice TG demonstraram associações significativas com pacientes com AOS em comparação com o grupo de controle ( *p* = 0,001). Além disso, os valores médios do índice de dessaturação de oxigênio (IDO), IAH, saturação mínima de oxigênio e porcentagem de tempo total de sono com saturação abaixo de 90% demonstraram diferenças estatisticamente significativas entre os grupos de índice TG ( *p* = 0,001; *p* = 0,001; *p* = 0,001; *p* = 0,003). O valor de corte ideal do índice TG para prever a AOS foi de 8,615 (AUC=0,638, IC de 95% = 0,606–0,671, *p* = 0,001). Na análise de regressão logística multivariada, após o ajuste para idade, sexo e índice de massa corporal, o índice TG foi independentemente associado a pacientes com AOS.

**Conclusão** O índice TG está independentemente associado a um maior risco de AOS. Isso indica que este índice, um marcador de gravidade da doença, pode ser usado para identificar pacientes com AOS grave em listas de espera para polissonografia.

## INTRODUCTION

Obstructive sleep apnoea (OSA) is a common disorder characterized by recurrent episodes of upper airway obstruction. Although the worldwide prevalence of OSA is 3–7% in men and 2–5% in women, its incidence has increased because of the obesity pandemic. 1 Recent studies have revealed that the prevalence of moderate to severe OSA is 49% in men and 23% in women. 2 Full-night polysomnography (PSG) remains the current gold standard diagnostic tool for OSA. However, the limited number of sleep laboratories and increased disease awareness have resulted in inadequate capacity to meet the growing demand.

The triglyceride–glucose (TG) index, a significant marker of insulin resistance, is calculated using fasting glucose and triglyceride levels 3 ; it is closely related to the homeostasis model assessment of insulin resistance, a well-known marker of insulin resistance. 4 However, it is an easier, quicker, and cheaper biomarker that can be measured without the need to assess insulin levels. A meta-analysis of 13 cohorts including 70,380 cases demonstrated a significant correlation between the TG index and type 2 diabetes mellitus. 5 Despite a standardized definition for insulin resistance, there is currently no standardized TG index cutoff value. 3

OSA is commonly associated with type 2 diabetes mellitus, obesity, and cardiac disease 6 ; intermittent hypoxia and sleep restriction, deprivation, and fragmentation contribute to the development of these diseases. Several studies have investigated the association between OSA and type 2 diabetes mellitus and have revealed that these conditions share common risk factors and exhibit a bidirectional relationship. 7 Moreover, OSA can have adverse effects on lipid metabolism independent of the presence of visceral obesity. 8 Chronic intermittent hypoxia and dysfunction of the sympathetic system are the significant contributing factors to the association between OSA and dyslipidemia; this strong association and the cause-effect relationship between OSA and dyslipidemia contribute to the development of atherosclerosis and end-organ damage. 8 9 Therefore, the TG index can be utilized as a biomarker for screening in clinical practice to identify patients at higher risk. Patients with clinical symptoms of OSA and a higher TG index could undergo PSA earlier than they otherwise would.

Only a few studies with small sample sizes have been conducted in different ethnic groups to determine the association between the TG index and OSA. Therefore, we investigated the association between the TG index and OSA severity and other sleep parameters in patients without diabetes mellitus, obesity, or metabolic syndrome. In addition, we determined a TG index cutoff value to identify severe OSA patients on waiting lists for PSG.

## METHODS

This retrospective cohort study included 6,173 patients with OSA suspicion between 1 January 2011 and 1 January 2020. The study was conducted in accordance with the Declaration of Helsinki. Our ethics committee approved the study protocol, and informed consent was obtained from all patients (Date: 25.02.2021/No: 90).

**Figure 1:**
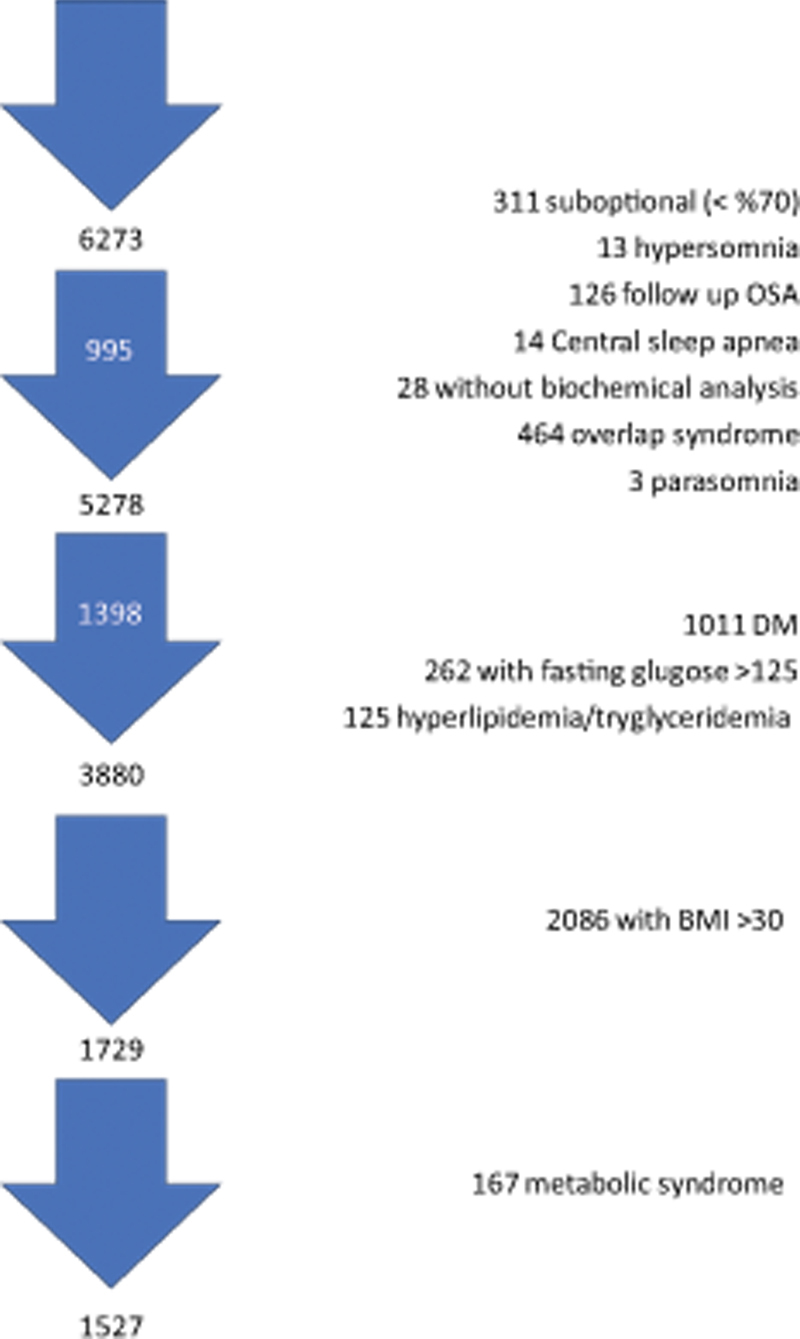
Flow chart of the study.

In total, 311 patients with suboptimal PSG report (sleep efficiency < 70%), 13 with hypersomnia, 3 with parasomnia, 464 with overlap syndrome, and 14 with central sleep apnoea; 26 patients who were previously diagnosed with OSA and were in follow-up; and 28 patients whose biochemical analyses could not be obtained were excluded. Moreover, 1,273 patients with diabetes mellitus, 125 with hyperlipidemia, 2,086 with body mass index [BMI] ≥ 30 kg/m ^2^ , and 267 with metabolic syndrome, as defined by the NCEP ATP III criteria, 10 were excluded. After applying the exclusion criteria, 1,527 patients were included in the final analysis. Figure 1 illustrates the flow chart of the study.

Baseline demographic data included age, sex, smoking history, medication use, comorbidities, BMI, PSG parameters, complete blood count, and biochemical data (levels of fasting blood glucose, triglycerides, high-density and low-density lipoproteins, and C-reactive protein).

Venous blood samples were collected following an overnight fast of ≥ 8 hours and analyzed within 1 hour. Complete blood cell counts were determined using a Sysmex XT4000i (Sysmex Corp., Kobe, Japan). Biochemical parameters were measured using a Beckman Coulter AU 2700 (Olympus, Tokyo, Japan). The TG index was calculated using the following formula: In(fasting triglyceride [mg/dL] × fasting glucose [mg/dL]/2).

Overnight PSG was performed using the Embla A-10 (Embla, Medcare Flaga, Reykjavik, Iceland) data acquisition and analysis system. Physiological signals were monitored via electroencephalography, electrooculography, electromyography, respiratory inductive plethysmography, piezoelectric sensors, nasal pressure cannula (Medcare Flaga), pulse oximetry, and electrocardiography (lead II). Sleep stages, arousals, and respiratory events were scored by a skilled pulmonary physician using the Somnologica Studio software package (Medcare Flaga), according to the American Academy of Sleep Medicine scoring manual. The PSG parameters recorded included total sleep time (TST), sleep period time (SPT), sleep efficiency (sleep%; TST/SPT), percentage of sleep stages, minimal oxygen saturation, apnoea-hypopnea index (AHI), oxygen desaturation index (ODI), and the TST percentages with saturation below 90% (TST90%).

Statistical analyses were performed using SPSS (version 25.0; IBM, Armonk, NY, USA). Categorical variables are presented as numbers (percentages), whereas continuous variables are presented as means ± standard deviations. For normally distributed data, the Student's *t* -test was used to compare the two groups. The Mann-Whitney U test was used for non-normally distributed data. The one-way analysis of variance test was used to compare three or more groups. Pearson's and Spearman's correlation tests were used as appropriate. The χ ^2^ test or Fisher's exact test was used to analyze categorical variables. Receiver operating characteristic curve analyses were used to determine an optimal cutoff point. Univariate logistic regression analyses were conducted to evaluate independent risk factors for OSA. Significant parameters identified in univariate analysis were included in multivariate logistic regression analysis. To avoid multicollinearity, only one parameter from the multiple clinical parameters was included in the analysis. P-values < 0.05 were considered statistically significant.

## RESULTS

We retrospectively evaluated 6,273 patients with OSA suspicion between 1 January 2011 and 1 January 2020, of which 1,527 were included. Most patients were male (81.5%) with a mean age of 43.99 ± 11.11 (15–90) years.

Based on the AHI, 1,174 patients (76.9%) were diagnosed with OSA, and 353 (23.1%) were included in the control group. OSA patients were predominantly older males and had a longer history of smoking, a higher proportion of ischemic heart disease, and higher values for BMI, AHI, ODI, TST90%, waist/hip and neck/hip ratios, and waist, neck, and hip circumferences.

Among OSA patients, 494 (32.4%) were classified as mild (AHI = 5–14.9), 359 (23.5%) as moderate (AHI = 15–29.9), and 321 (21.0%) as severe (AHI > 30). The demographic and clinical parameters of the patients are summarized in Table 1 .

**Table 1:** Demographical and clinical characteristics of patients with severity of OSA

		No OSA ( *n* = 353)	Mild OSA ( *n* = 494)	Moderate OSA ( *n* = 359)	Severe OSA ( *n* = 321)	*P*
Age		39.51 ± 11.39	44.25 ± 10.38	46.28 ± 10.46	45.97 ± 11.19	0.001*
Gender (male %)		224 (63.5%)	399 (80.8%)	324(90.3%)	298 (92.8)	0.001*
BMI (kg/m ^2^ )		25.36 ± 2.86	26.53 ± 2.22	26.94 ± 1.91	27.34 ± 2.02	0.001*
Waist Circumference (cm)		87.72 ± 9.68	92.92 ± 7.91	94.92 ± 6.95	96.34 ± 7.36	0.001*
Hip Circumference (cm)		97.52 ± 7.64	100.01 ± 6.44	99.74 ± 7.38	100.63 ± 5.38	0.001*
Neck Circumference (cm)		35.27 ± 3.41	36.75 ± 3.19	37.67 ± 2.88	38.13 ± 3.09	0.001*
WHR		0.903 ± 0.123	0.930 ± 0.067	0.964 ± 0.272	0.958 ± 0.064	0.001*
NHR		0.208 ± 0.017	0.214 ± 0.018	0.218 ± 0.016	0.220 ± 0.018	0.001*
Smoking status, n (%)	Active smoker	155 (44.2%)	188 (38.1%)	139(38.7%)	142 (44.2%)	0.007*
	Never smoker	152 (43.3%)	193 (39.1%)	133 (37.0%)	104 (32.4%)	
	Ex smoker	44 (12.5%)	113 (22.9%)	87(24.2%)	75 (73.4%)	
Cigarette pocket/year		15.50 ± 10.33	16.99 ± 9.99	18.02 ± 12.70	18.14 ± 10.55	0.051
Hypertension (%)		23 (6.5%)	33 (6.7%)	36 (10.0%)	41 (12.8%)	0.007*
IHD (%)		2 (0.6%)	14 (2.8%)	14 (3.9%)	12 (3.7%)	0.026*
CHF(%)		8 (2.3%)	20 (4.0%)	20 (5.6%)	11 (3.4%)	0.142
Total cholesterol (mg/dL)		198.14 ± 42.32	210.70 ± 41.03	213.61 ± 42.85	214.77 ± 41.01	0.001*
HDL cholesterol level (mg/dL)		49.50 ± 12.06	47.56 ± 11.21	45.56 ± 10.02	44.26 ± 9.37	0.001*
LDL cholesterol level (mg/dL)		124.31 ± 35.89	134.15 ± 35.66	156.54 ± 37.15	136.93 ± 35.26	0.001*
Triglyceride level (mg/dL)		122.79 ± 72.10	145.35 ± 96.20	159.25 ± 107.39	167.18 ± 99.39	0.001*
Glucose level (mmol/L)		92.92 ± 9.14	95.16 ± 8.88	95.68 ± 9.15	97.30 ± 8.88	0.001*
TyG		8.51 ± 0.49	8.69 ± 0.53	8.78 ± 0.52	8.86 ± 0.51	0.001*
CRP (mg/dL)		2.80 ± 4.89	3.05 ± 4.92	3.21 ± 3.76	4.75 ± 8.02	0.001*
ESS		8.24 ± 4.17	8.71 ± 4.54	8.45 ± 4.28	9.64 ± 4.85	0.001*
Total sleep time (min)		402.87 ± 40.52	407.98 ± 38.27	403.49 ± 42.27	386.89 ± 62.73	0.001*
Sleep (%)		86.95 ± 7.16	87.77 ± 7.21	87.31 ± 7.25	87.16 ± 7.22	0.397
REM (%)		19.66 ± 5.60	20.27 ± 5.40	19.27 ± 5.87	17.08 ± 6.34	0.001*
AHI (1/h)		2.30 ± 1.46	9.09 ± 2.70	20.99 ± 4.04	48.31 ± 15.34	0.001*
ODI (1/h)		2.26 ± 5.34	6.92 ± 4.39	16.87 ± 6.02	41.44 ± 16.67	0.001*
TST 90% (min)		3.25 ± 23.85	6.90 ± 28.82	11.14 ± 24.95	45.45 ± 56.21	0.001*
Min Sat02 (%)		91.06 ± 3.14	87.59 ± 4.40	84.85 ± 5.52	78.72 ± 8.39	0.001*

Abbreviations: AHI, apnea hypopnea index; BMI, body mass index; CHD, congestive heart disease; ESS, epworth sleep scale; IHD, ischemic heart disease; Min Sat O _2_ %, minimum oxygen saturation percentage; NHR, ratio of neck to hip circumference; ODI, oxygen desaturation index; TST90%, time spent with O _2_ under 90%; TyG, triglyceride glucose index; WHR, ratio of waist to hip circumference.

Notes: ^†^ mean ± SD.

*: *p* < 0.05.

The TG index demonstrated significant correlations with various anthropometric and sleep-related parameters; it was positively correlated with BMI (r = 0.21, *p* < 0.001), waist/hip (r = 0.22, *p* < 0.001) and neck/hip ratio (r = 0.25, *p* < 0.001), and waist (r = 0.22, *p* < 0.001), hip (r = 0.10, *p* < 0.001), and neck circumference (r = 0.29, *p* < 0.001). Furthermore, it showed positive associations with AHI (r = 0.23, *p* < 0.001), ODI (r = 0.25, *p* < 0.001), and TST90% (r = 0.06, *p* < 0.01); however, it correlated negatively with minimum oxygen saturation (r =  −0.14, *p* < 0.001).

**Figure 2:**
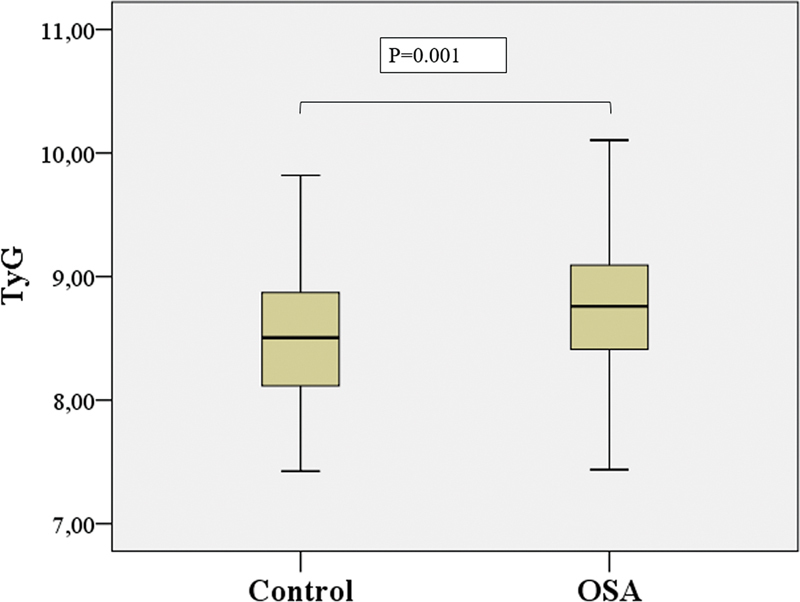
Comparison of TyG values of patients with and without diagnosis of OSA by plot graph.

**Figure 3:**
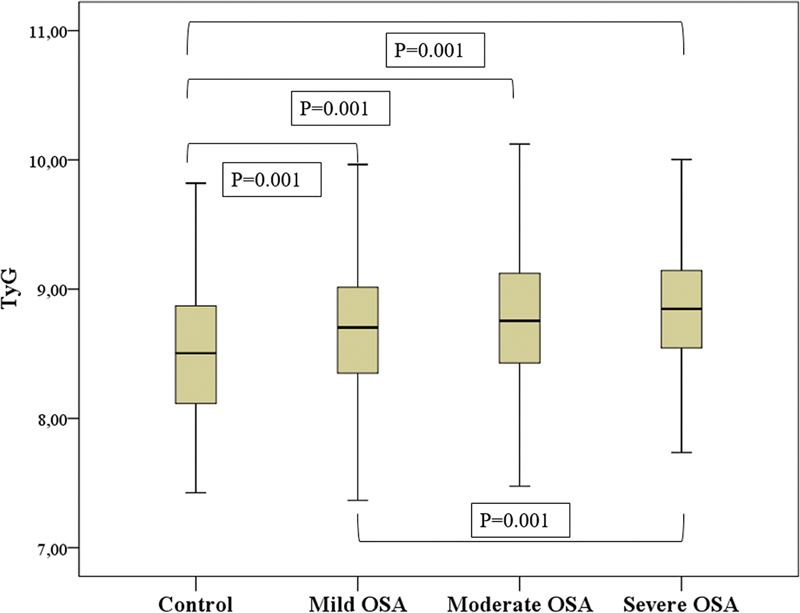
Comparission of TyG values of patients with severity of OSA by plot graph.

The mean TG index of the OSA and control groups was 8.77 and 8.51, respectively (mean difference = 0.25, 95% confidence interval [CI] = 0.18–0.31, *p* < 0.001, Figure 2 ). The TG index values in the control group significantly differed from all three OSA severity groups. Moreover, no significant differences were observed between the moderate and mild or severe OSA groups. However, a significant difference was found between the mild and severe OSA groups ( *p* = 0.001, Figure 3 ).

**Figure 4:**
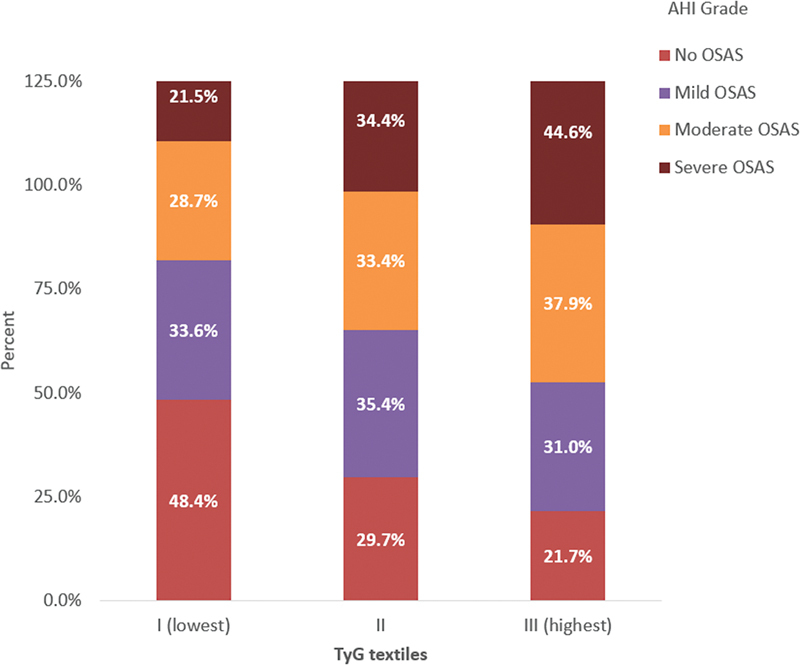
Composition of prevalence and severity of OSA according to TyG index textiles.

**Figure 5:**
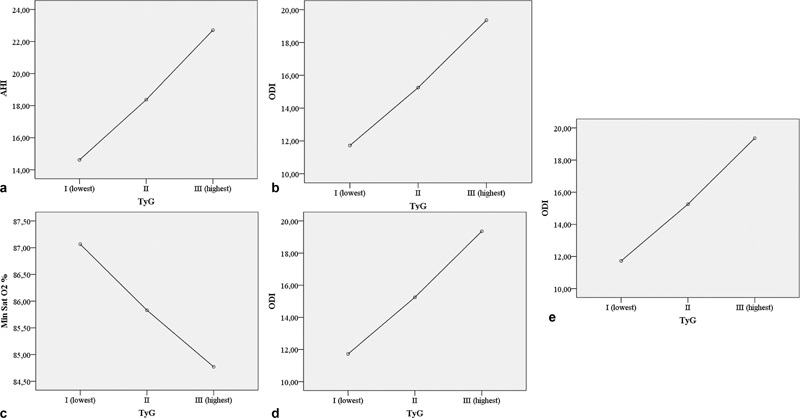
(a) Means plot graphs of ODI; (b) Means plot graphs of AHI (c) Means plot graphs of the minimum Sat O2 (d) Means plot graphs of TST %90. time in minutes determined according to TyG index tertiles (p: 0.001; p:0.001; p:0.001; p:0.003) (e) Optimal TyG index cut-off value for predicting OSA.

The TG index values were categorized into group 1 (7.28–8.47), group 2 (8.47–8.91), and group 3 (8.91–10.79). In the control group, 48.4%, 29.7%, and 21.8% of patients were categorized in the first, second, and third groups, respectively. In the OSA group, 28.8%, 34.4%, and 36.8% of patients were categorized in the first, second, and third groups, respectively. The number of OSA cases was evaluated based on the TG index groups; a statistically significant association was found between OSA and the third group ( *p* = 0.001). The prevalence and severity of OSA, as determined by the TG index groups, demonstrated significant associations ( Figure 4 ). In addition, the mean values of ODI, AHI, minimum Sat O2%, and TST%90 were statistically significant across the TG index groups ( Figure 5 ).

The optimal TG index cutoff value to predict OSA was determined to be 8.615 using receiver operating characteristic analysis (area under the curve [AUC] = 0.638, 95% CI = 0.606–0.671, *p* = 0.001); at this cutoff value, the sensitivity and sensitivity were 59.9% and 59.8%, respectively. In addition, the AUC for the TG index to predict moderate and severe OSA was 0.610 (95% CI = 0.581–0.638, *p* < 0.001), and for severe OSA alone was 0.607 (95% CI = 0.574–0.641, *p* < 0.001).

When the receiver operating characteristic analyses were conducted according to sex, the TG index cutoff value for predicting OSA in males was 8.689 (AUC = 0.606, 95% CI = 0.565–0.647), with a sensitivity and specificity of 42.4%; moreover, the cutoff value for predicting OSA in females was 8.446 (AUC = 0.617, 95% CI = 0.551–0.682), with a sensitivity and specificity of 40.3% and 40.5%, respectively.

In multivariate logistic regression analysis, after adjusting for age, sex, BMI, and smoking, the TG index was independently associated with OSA severity (odds ratio = 1.58; 95% CI = 1.206–2.073, *p* = 0.001).

## DISCUSSION

We investigated the association between the TG index and OSA patients without diabetes mellitus, obesity, or metabolic syndrome in a Caucasian population. There was a significant association between the index and OSA, and the index significantly correlated with disease severity.

Several studies have demonstrated that the TG index is significantly associated with OSA and its severity. 11 12 However, those studies used objective sleep tests instead of PSG. As a result, they lacked sleep parameters and data on sleep stages, limiting their ability to evaluate the relationship between the index and these specific sleep parameters. Furthermore, those studies did not exclude OSA patients with diabetes mellitus and obesity. Although multiple studies conducted on Asian populations have used PSG, their findings are not directly applicable to the general population because of the influence of genetic factors on OSA and its metabolic consequences. 13

OSA patients without diabetes mellitus or obesity are significantly associated with higher TG index values. 14 Although chronic intermittent hypoxemia can lead to insulin resistance in OSA patients, other underlying mechanisms, such as sleep fragmentation, deprivation, or restriction, can also contribute. 15 16 Bikov et al. 13 reported a lack of significant correlations between TG index values or lipid profiles and the markers of sleep quality in OSA patients. However, we found a significant association between TG index values and sleep parameters. Unlike Bikov et al., 13 we excluded patients who received hyperlipidemic treatment, as it has been shown to affect sleep quality.

Among multiple studies conducted in China, Korea, Italy, and Switzerland, only one excluded OSA patients with obesity and diabetes mellitus; that study demonstrated a significant increase in TG index values in severe OSA patients compared with controls, with minimal heterogeneity. 11 12 13 14 Although ethnicity can potentially influence the association between TG index values and OSA severity, similar results were observed in a Turkish population.

Age is recognized as a significant predictor of OSA. However, we did not find a significant association between age and TG index values. 17 Similar to previous studies, we observed higher TG index values in male patients with OSA. 11 12 13 14 In addition, TG index cutoff values to predict OSA were higher in males than females (8.69 versus 8.45); these findings are consistent Wang et al. (8.69 versus 8.24). 18 We observed a significant correlation between the TG index values and hypertension, consistent with previous studies 19 20 ; however, we did not find significant associations between the TG index values and cardiovascular diseases. Previous studies have revealed a correlation between obesity and TG index values. 21 Similar to Bikov et al., 14 we also noted an association in individuals with BMI < 30.

AHI is commonly used to classify the severity of OSA. However, other parameters revealed during PSG evaluations can help determine the disease severity. 22 Our study revealed that the TG index correlates significantly with AHI, ODI, TST90%, and minimum oxygen saturation, consistent with previous studies. 14

After adjusting for risk factors associated with OSA, TG index values were significantly higher in OSA patients than in controls ( *p* = 0.02). Higher TG index values are significantly associated with disease severity in non-obese, non-diabetic OSA patients. 14 After adjusting for age, sex, BMI, and smoking, we found that the TG index values were significantly related to the disease severity in OSA patients who did not have obesity, diabetes mellitus, or metabolic syndrome.

This study had some limitations. First, due to the retrospective design, we were unable to gather information about the long-term dietary habits of the patients, which could influence fasting glucose levels. Second, there was an imbalance in the sex distribution, with more males than females being referred to sleep clinics during the study period. Third, we could not include other insulin-related parameters to compare their diagnostic power with the TG index. Fourth, our control group consisted of habitual snorers who required PSG evaluation; several studies have suggested that habitual snoring is associated with insulin dysregulation, even in non-obese individuals. 23 Therefore, the inclusion of a control group without OSA symptoms could provide a more pronounced diagnostic effect. Conversely, our study also had some strengths. First, it had a large sample size and collected comprehensive PSG data from all patients, including full-night attended 16-channel recordings. Second, medication histories were conducted for each patient before the PSG examination upon admission to the sleep laboratory.

In conclusion, an increased TG index is independently associated with a higher risk for OSA in nondiabetic, nonobese, nonmetabolic syndrome patients. We observed significant associations between the index and various sleep parameters, including AHI, the mean value of ODI, minimum oxygen saturation, and TST90%. These findings indicate that the TG index, a marker for disease severity, can be used to identify severe OSA patients on waiting lists for PSG. Prospective clinical trials are needed to evaluate the effectiveness of the TG index in routine clinical practice.
